# Factors associated with work engagement among specialist nurses in china: a cross-sectional study

**DOI:** 10.1186/s12912-024-02012-w

**Published:** 2024-05-28

**Authors:** Lichun Xu, Liyu Lin, Aixuan Guan, Qingqing Wang, Feng Lin, Weicong Lin, Jing Li

**Affiliations:** 1 Zhongshan Hospital Xiamen University, Xiamen, China; 2 Xiamen Nursing Quality Control Centre, Xiamen, China; 3 School of Nursing, Fujian University of Traditional Chinese Medicine, Fuzhou, China

**Keywords:** Specialist nurses, Work engagement, Nursing management, China

## Abstract

**Background:**

The positive impacts of work engagement among specialist nurses on retention, organizational commitment, and quality of care are well-documented. However, there is a lack of research on the specific differences in work engagement among specialist nurses. Therefore, the purpose of this study is to assess the level of work engagement among specialist nurses in China and identify its influencing factors.

**Methods:**

A descriptive cross-sectional study was conducted in China from April to July, 2023, with 724 nurses selected from 22 hospitals through convenience sampling involved. The survey was conducted by using self-administered general information questionnaires and work engagement scales. Questionnaire Star was employed as the online data collection tool. The collected data was analyzed by using descriptive statistics and stepwise regression analysis to draw meaningful conclusions from the study.

**Results:**

Among specialist nurses in Xiamen, China, who had a response rate of 97.10%, an average work engagement score is 140.35 (SD=18.17), with the highest score for the work attitude at 4.65 (SD=0.52) and the lowest score for the work recognition at 4.09 (SD=0.85). It was shown through regression analysis that factors such as career satisfaction, involvement in challenging case discussions, marital status, gender, presence of promotion advantage and title accounted for 14.5% of the total variance in the model and were significant explanatory variables that could predict work engagement.

**Conclusion:**

It is shown that specialist nurses in Xiamen, China have a high level of work engagement. It is imperative for nursing managers to prioritize the work engagement of specialist nurses, provide the specialist nurses with ample development opportunities and room for growth, and effectively promote the overall development of specialist nurses by improving work engagement in various aspects.

**Supplementary Information:**

The online version contains supplementary material available at 10.1186/s12912-024-02012-w.

## Introduction

Work engagement is a working state in which individuals exhibit high levels of vitality, dedication and concentration, which is shown through their energetic approach to tasks, their ability to adapt to challenges, their deep appreciation for the significance and motivation provided by work, and their unwavering commitment to the tasks at hand [1]. Work engagement is considered as a central factor in enhancing competitiveness within healthcare systems, which could provide high-level work engagement to patients [2]. Nurses, being the largest group of healthcare workers, are recognized for their essential role in providing care and support to patients, whose engagement is a key component of their individual potential and work indicators [3]. Specialist nurses (SN), being registered nurses who possess the expertise to effectively apply nursing knowledge and advanced technology in order to deliver specialist services to their patients, have a high level of proficiency that plays a crucial role in enhancing nursing competence and increasing the overall competitiveness of hospitals [4–6]. Therefore, by elucidating influencing factors of the work engagement of specialist nurses, a foundational reference is provided for further training the specialist nurses and enhancing the work motivation of the specialist nurses.

## Background

The United States was a pioneer in the development of specialist nursing, which is typically categorized into two levels: primary specialist nurses and advanced practice nurses (APNs). An APN is a registered nurse with a master's degree who possesses extensive theoretical knowledge, advanced decision-making skills, clinical proficiency and abilities in early identification and management of potential or existing health problems [7]. As specialist nursing continues to advance in China, a specialist nurse is defined as a registered nurse capable of delivering advanced nursing services to patients within a specific field by skillfully applying nursing knowledge and clinical skills under the support of specialist training courses, clinical practice and successful completion of qualification exams [4]. Compared to general nurses in China, specialist nurses focus on specific areas with more in-depth professional knowledge and skills, and mainly play a role in teaching, management, scientific research, discussion of challenging medical cases, outpatient clinics, consultations, and so on. Currently, only a limited number of specialties offer dedicated nursing clinics [8], who are staffed as specialist nurses but currently have no authority to prescribe medication, and are also involved in specific aspects of specialist nursing within their career scope. Despite advancements in specialist nursing in recent years, China lacks a standardized and perfect training and certification system for specialist nurses compared with other countries. There is a lack of unified selection criteria and entrance standards as well as full-time positions for specialist nurses, which hinders the abilities of the specialist nurses to fully utilize their skills in nursing clinics and research. The implementation of specialist nursing clinics and scientific research in China remains inadequate [8, 9]. It is indicated that specialist nursing in China is currently in the stage of exploration and development, and there is still much progress to be made in this field within the country.

Specialist nurses perform in a variety of aspects, with a strong emphasis on leadership and education, including providing information, advice and support, conducting nursing management and participating research, teaching and service development [10, 11]. With the ongoing professional development of the nursing team and the increasing demand of patients for health services, the importance of nursing services has continued to expand, which has led to a significant evolution in the role of specialist nurses. Specialist nurses are now focusing more on specialization and can practice in various settings, including subspecialty outpatient practices and inpatient units [12]. As specialist nursing practice continues to be more specialized, the establishment of a specialist nursing team is crucial for the advancement and quality enhancement of the nursing profession, and work engagement becomes the core competitiveness of hospitals [2]. Work engagement significantly influences nurses' physical and psychological health as well as their overall work performance. Previous studies on nurses have shown the beneficial effects of work engagement on work satisfaction, employee retention, organizational commitment and the delivery of high-quality patient care [13, 14]. Hence, it is imperative to study work engagement and its associated factors among specialist nurses in consideration that it allows nurse managers to pinpoint and provide assistance to nurses experiencing low work engagement, ultimately safeguarding the quality of nursing care. Despite the pivotal role that work engagement plays in maintaining positive outcomes for both nurses and patients, there remains a lack of research on this topic specifically pertaining to specialist nurses. It has been shown by studies [15] that Spanish nurses exhibit high levels of work engagement, while it has also been shown by another study [16] that Chinese nurses exhibit moderate levels of work engagement. However, it should be noted that specialist nurses differ from general nurses especially in the aspects of nursing workload, psychological stress and practice environment. Therefore, there is a need for further investigation to determine the current status of work engagement among specialist nurses and to identify potential influencing factors, thereby providing valuable evidence for interventions.

Several scholars have delved into the work engagement of specialist nurses in various fields such as oral care [17], paediatric [18], psychiatry [19]. A study by Qin Hui [20] focused on the situation of specialist nurses in Jiangsu Province showed that the work engagement among provincial specialist nurses in Jiangsu Province was at a medium to high level, with influencing factors including gender, age, type of specialty, mode of appointment, type of specialized posting, specialized outpatient clinics and the status of thesis publication. This paper aims to investigate the current situation of specialist nurses in Xiamen and identify the influencing factors, in order to ultimately achieve the goal of providing a reference for targeted interventions.

**Fig. 1:**
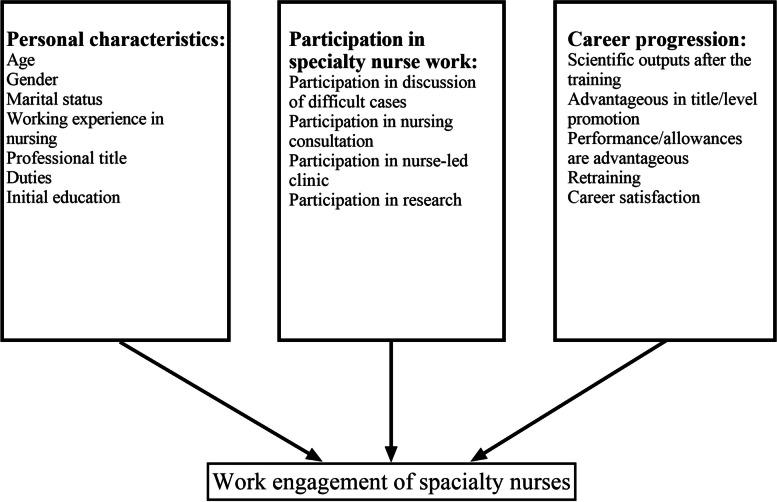
Conceptual framework of this study

The purpose of this study was to investigate the status and influencing factors of work engagement among specialist nurses in hospital settings. Specifically, we analyzed differences in personal characteristics, involvement in specialist nursing tasks and career progression in relation to work engagement among specialist nurses. A conceptual framework illustrating pathways to varying levels of work engagement was used in this study for determining a range of factors including personal characteristics (such as age, gender, marital status, nursing experience, professional title, duties, and education level), involvement in specialist nursing tasks (such as involvement in challenging case discussions, involvement in nursing consultations, involvement in nurse-led clinics, and involvement in research), and career progression (including post-training scientific contributions, title/level advancement , performance/allowance incentives, retraining opportunities, and career satisfaction) (see Fig. 1). The results of this study may offer valuable information to nurse managers to develop targeted strategies for enhancing the work engagement of clinical nurses.

## Methods

### Design

This cross-sectional study was conducted from April to July 2023 in 22 hospitals located in Xiamen, China, which adhered to the “Guidelines for Strengthening the Reporting of Observational Studies in Epidemiology (STROBE) [21] ” (see Appendix) to ensure the robustness and transparency of the research methodology.

### Participants and settings

A convenience sampling method was utilized to select a total of 724 specialist nurses from all hospitals in Xiamen from April to July 2023, and we also recruited nurses specializing in medical, surgical, ICU, emergency and Chinese medicine fields. Informed consent was obtained from each participant, with inclusion criteria as follows: nurses should hold certificates of nursing practice and specialist nurse qualification in their respective specialty, and have completed systematic training and passed the examination. Exclusion criteria included unwillingness to participate in the study or absence for various reasons during the investigation.

This study utilized the Chinese version of the “Specialist Nurse Work Engagement Scale”, which consists of 32 items. The sample size was determined using the Kendall M sample size formula, which recommend to involve at least 10 times the number of questionnaires, including 20% for invalid responses (*n*=384). As a result, the calculated sample size was initially set at a minimum of 388. However, the final sample size for this study ended up being 703 participants.

### Survey tools

#### Demographic characteristics

A self-administered questionnaire was carefully crafted to gather sociodemographic information from participants, including a range of general information such as hospital level, department, age, gender (female and male), marital status (married and unmarried), working experience in nursing, professional titles (nurse practitioner, head nurse, associate chief nurse or above), duties (clinical nurse, chief clinical instructor, supervisor nurse), education levels (technical secondary school, junior college, bachelor's degree and above), involvement in challenging case discussions (yes or no), involvement in nursing consultations (yes or no), involvement in nurse-led clinics (yes or no), involvement in research, post-training scientific contributions (yes or no), title/level advancement, performance/allowance incentives (yes or no), retraining opportunities and career satisfaction (yes or no).

#### Work engagement

Work engagement was assessed using the Chinese version of the “Specialist Nurse Work Engagement Scale” [22], which is a self-reported tool consisting of 32 items categorized into five domains: work attitude (8 items), work values (6 items), work recognition (4 items), work motivation (9 items) and work concentration and enthusiasm (5 items). The frequency of work engagement of the specialist nurses was assessed using a 5-point Likert scale, with the score from 1 (never) to 5 (all the time), in which a higher score indicated a higher level of work engagement. Additionally, the Cronbach's alpha coefficients ranged from 0.827 to 0.905 in different dimensions and ranged from 0.766 to 0.808 for half reliabilities. The I-CVI values of the scale ranged from 0.834 to 1.000, and the S-CVI value of the scale was 0.842.

### Data collection

In order to guarantee the accuracy and reliability of the online survey, we secured the endorsement and collaboration of the directors of nursing at each hospital. Prior to conduct the survey, we took measures to ensure that all participants had a thorough understanding of the specific inclusion and exclusion criteria in this study. After obtaining informed consents from all nurses, an electronic questionnaire was distributed to eligible nurses via the WeChat platform by the head nurses or relevant ward staff. Nurses were encouraged to complete the questionnaire during their breaks to ensure authenticity, rather than rushing through it during working hours. It was clearly communicated that involvement in the study was voluntary and nurses had the option to withdraw at any time. To maintain the quality and completeness of the questionnaire, a uniform guideline was utilized to introduce the purpose, significance, and method of completing the study. The questionnaire was designed to be completed anonymously, with the limit of one submission per IP address in order to ensure the authenticity and reliability of the data. All questions were mandatory and could only be submitted after all entries have been completed in order to ensure data integrity.

During the question collection phase, each questionnaire was reviewed individually to identify and remove any submissions that took less than 180 seconds to complete or had the same option selected for all items, which better ensured the quality of the data collected. Upon completion of data collection, two researchers conducted a thorough verification and collation of the data in duplicate to ensure accuracy and consistency.

### Data analysis

The data cleaning, statistical description and analysis in this study were conducted using SPSS software (version 26.0). Measurement data were reported as mean ± SD, while count data were presented as frequencies and percentages. Work engagement scores were compared across socio-demographic variables using t-tests and ANOVA. Variables having statistically significant differences in univariate analysis and work engagement were included in multiple linear stepwise regression analysis for further analysis. The significance level was set at α = 0.05, with *P* < 0.05 indicating statistically significant differences. Before regression analyses, it was confirmed that the data met the basic assumptions of regression, including normal distribution of residuals, linearity, Chi squareness of variance, and multicollinearity.

### Ethical considerations

In this study, all methods were performed according to the relevant guidelines and regulations. The study was approved by the Ethics Committee of Zhongshan Hospital Xiamen University (2023-176). All procedures were in accordance with the ethical standards of the responsible committee on human experimentation and with the Declaration of Helsinki. Data collection was initiated only after obtaining informed consents from all participants, and all information was gathered anonymously.

## Result

In this study, a total of 724 questionnaires were distributed, out of which 703 effective questionnaires were successfully retrieved, giving an effective response rate of 97.10%.

### General characteristics of work engagement

The score for work engagement of the specialist nurses was 140.35 (SD = 18.17), with the highest score for the work attitude of 4.65 (SD = 0.52) and the lowest score for the work recognition of 4.09 (SD = 0.85), see Table 1. Notably, those in the item “I believe that I will always be able to maintain a prudent attitude in my future work” exhibited the highest score of 4.75 (SD = 0.542) within the dimension of work attitude. Conversely, those in the item “I get to the point where I forget myself when I'm working” exhibited the lowest score of 3.94 (SD = 0.876) within the dimension of work concentration and enthusiasm. See Table 2 for details of the three highest scores and the three lowest scores.

**Table 1:** Score for work engagement of specialist nurses (*n* = 703)

Variable	Scores M (SD)	Average score for each item M (SD)
Scale for work engagement of specialist nurses	140.35 (18.17)	4.39 (0.57)
Work attitude	37.19 (4.16)	4.65 (0.52)
Work values	26.53 (4.12)	4.42 (0.69)
Work recognition	16.37 (3.40)	4.09 (0.85)
Work motivation	39.20 (5.61)	4.36 (0.62)
Work concentration and enthusiasm	21.06 (3.32)	4,21 (0.66)

**Table 2:** Items with top three and bottom three scores for work engagement of specialist nurses (*n*=703)

Item	Scores M (SD)	Dimension
Items with top three scores		
I believe that I can always maintain a prudent attitude in my future work as well	4.75 (0.542)	Work attitude
I work with a strong sense of responsibility and mission	4.71 (0.570)	Work attitude
I will actively cooperate with the rest of the team in the performance of the position	4.69 (0.572)	Work attitude
Last 3 items		
I get to a point where I forget myself when I'm working	3.94 (0.876)	Work concentration and enthusiasm
I will actively conduct nursing research and innovate ideas and methods of work	3.96 (0.961)	Work motivation
Specialist nursing can be highly recognized by the community	3.97 (0.968)	Work recognition

### Demographic information and level of the main variables

Univariate analysis showed that various factors such as age, gender, marital status, years of work experience, professional title, duties, initial qualification, post-training research achievements, involvement in challenging case discussions, involvement in nursing consultations, involvement in specialist nursing clinics, involvement in teaching and research activities, post-training scientific contributions, title/level advancement, performance/allowance incentives, retraining opportunities and career satisfaction significantly influenced work engagement of the specialist nurses. See Table 3 for further details.

**Table 3:** Study of scores for work engagement based on different characteristics (*n* = 703)

Variable	Category	n (%)	Mean (SD)	F/t	P
Age (years)	≤35	295 (42.0%)	138.54 (18.97)	-2.257	0.024
	>35	408 (58.0%)	141.66 (17.47)		
Gender	Male	56 (8.0%)	135.30 (22.09)	-2.173	0.030
	Female	647 (92.0%)	140.79 (17.74)		
Marital status	Married	602 (85.6%)	141.25 (18.09)	3.251	0.001
	Unmarried	101 (14.4%)	134.95 (17.80)		
Working experience in nursing (years)	≤15	403 (57.3%)	138.87 (18.58)	-2.539	0.011
	>15	300 (42.7%)	142.34 (17.44)		
Professional title	Nurse practitioner	132 (18.8%)	136.64 (21.85)	5.962	0.003
	Nurse-in-charge	469 (66.7%)	140.42 (17.60)		
	Associate chief nurse or above	102 (14.5%)	144.84 (14.19)		
Duties	Clinical nurse	472 (67.1%)	139.75 (18.64)	3.557	0.029
	Chief clinical instructor	96 (13.7%)	138.25 (19.10)		
	Supervisor nurse	135 (19.2%)	143.94 (15.24)		
Initial education	Technical secondary school	162 (23%)	143.85 (17.02)	3.922	0.020
	Junior college	244 (34.7%)	139.29 (19.35)		
	Bachelor's degree and above	297 (42.2%)	139.32 (17.59)		
Post-training scientific contributions	No	467 (66.4%)	138.82 (19.33)	3.432	0.001
	Yes	236 (33.6%)	143.39 (15.20)		
Involvement in challenging case discussions	No	464 (66%)	138.73 (19.26)	-3.554	<0.001
	Yes	239 (34%)	143.49 (15.41)		
Involvement in nursing consultations	No	533 (75.8%)	138.96 (18.92)	-4.108	<0.001
	Yes	170 (24.2%)	144.71 (14.79)		
Involvement in specialist nursing clinics	No	568 (80.8%)	139.44 (18.10)	-2.755	0.006
	Yes	135 (19.2%)	144.21 (18.02)		
Involvement in teaching	No	345 (49.1%)	138.95 (19.42)	-2.013	0.045
	Yes	358 (50.9%)	141.70 (16.79)		
Involvement in Research	No	625 (88.9%)	139.76 (18.34)	-2.444	0.015
	Yes	78 (11.1%)	145.08 (16.06)		
Title/level advancement	No	368 (52.3%)	137.98 (16.62)	3.659	<0.001
	Yes	335 (47.7%)	142.96 (19.43)		
Performance/allowance incentives	No	607 (86.3%)	139.64 (17.33)	2.635	0.009
	Yes	96 (13.7%)	144.88 (22.37)		
Specialist nurses' career satisfaction	Rather unsatisfactory	239 (34%)	132.76 (18.84)	35.947	<0.001
	Relatively Satisfactory	464 (66%)	144.26 (16.52)		
Retraining	No	525 (74.7%)	139.53 (18.04)	2.080	0.038
	Yes	178 (25.3%)	142.79 (18.37)		

### Multiple linear stepwise regression analysis of influencing factors of work engagement

Multiple linear stepwise regression analysis was conducted, incorporating variables that exhibited statistical significance in univariate analysis. The results (see Table 4) that career satisfaction, involvement in challenging case discussions, marital status, gender, possession of promotion advantage and job title emerged as significant predictors of work engagement of the specialist nurses (*R*^*2*^=0.155, adjusted *R*^*2*^=0.145,* F*=15.918, *P*<0.001).

**Table 4:** Multiple linear stepwise regression analysis of work engagement of specialist nurses

Variable	B	SE	β	*t*	*P*	VIF
Constant	116.018	3.181	——	36.476	<0.001	——
Relatively satisfactory (vs. Rather unsatisfactory) career development	11.574	1.366	0.302	8.473	<0.001	1.043
Involvement in challenging case discussions (vs. No)	3.465	1.355	0.090	2.556	0.011	1.027
Post-training scientific contributions (vs. No)	2.799	1.441	0.073	1.943	0.052	1.153
Married (vs. Unmarried)	4.012	1.892	0.078	2.120	0.034	1.097
Female (vs. Male)	6.67	2.365	0.099	2.820	0.005	1.022
Title/level advancement (vs. No)	3.227	1.294	0.089	2.493	0.013	1.041
Associate chief nurse or above (vs. Nurse practitioner)	7.24	2.467	0.140	2.935	0.003	1.880
Nurse-in-charge (vs. Nurse practitioner)	3.621	1.751	0.094	2.068	0.039	1.696

*Abbreviations*: *B* Partial Regression Coefficient, *SE* Standard Error, β Standard Regression Coefficient, *VIF* Variance inflation factor

*R*^2^=0.155, Adjust *R*^2^=0.145, *F*=15.918, *P*<0.001

## Discussion

The study investigated work engagement among specialist nurses in China, indicating that participants exhibited high levels of work engagement. However, it was found through a study [23] conducted in Hunan Province that hospital-based specialist nurses exhibited only moderate levels of work engagement on average. This difference may be attributed to variations in the scales used to measure work engagement. Wang et al [23] used a 9-item UWES questionnaire [24], which was different from that used in this study. Furthermore, the overall scores for work engagement of specialist nurses in Xiamen surpassed those of specialist nurses in Sichuan Province [3] and those of specialist nurses in Jiangsu Province [20] where the same tools were used, with the reason that the increasing emphasis had been placed by the government on the training and development of specialist nurses in recent years. The “Outline of China's Nursing Career Development Plan” underscores the importance of establishing a team of specialist nurses, enhancing specialist nurse training and improving the quality of specialist nursing care. Additionally, the geographical location of participants may have played a role in the results. Being a more developed city with ample medical resources, Xiamen likely fosters closer collaboration and support among nursing teams, thereby potentially boosting the level of work engagement of specialist nurses.

It was found through this study that the score for work engagement of nurses in China in the dimension of work attitude was highest with the possible reasons of comprehensive professional training and strong sense of professionalism and responsibility among specialist nurses. Conversely, the score in the dimension of professional recognition was lowest with the possible reason of absence of a standardized program for nurse deployment and management in China. Despite the increasing number of specialist nurses in China, research on their training remains limited. Currently, specialist nurses in China do not hold full-time positions or have explicit prescribing rights, leading to unclear job responsibilities and an inadequate remuneration system [25]. To address these challenges, hospitals and nursing managers should enhance the management system for specialist nurses, boost their professional identity, provide better support, optimize their work environment, establish a fair and effective incentive mechanism, foster a sense of achievement and ultimately increase their commitment to work, thereby ultimately contributing to the high-quality development of hospitals and specialist nursing teams.

Those in the item "I firmly believe that I possess the ability to uphold a cautious approach in my future endeavors" exhibited the highest score as the top-ranking response in the survey conducted among Chinese specialist nurses regarding their work engagement, with the possible reason closely linked to the importance of discretion in the field of nursing. Deliberateness, characterized by thoroughness and prudence in decision-making, is an essential trait for nurses. Given the demanding nature of nursing work, where nurses often have to navigate complex medical scenarios independently in a fast-paced environment, a sense of deliberateness is vital for specialist nurses [26]. Conversely, Those in the item "I achieve a state of selflessness in my work" exhibited the lowest score, possibly due to factors such as the hectic schedules that nurses often contend with. Specialist nurses frequently face heavy workloads, intricate medical scenarios and intense work rhythms, making it difficult for them to fully engage in their work. In addition, prolonged exposure to such pressures can result in fatigue, anxiety and other challenges which may in turn impact their commitment to work [27]. To further enhance work engagement of nurses, it is imperative to address issues such as workload management, and hospitals and nurse managers can alleviate workload pressures and help them to be more engaged by strategically planning nurses' tasks, streamlining workflows and providing necessary support and resources.

It is crucial for implementing interventions to improve work engagement by investigating the influencing factors of work engagement. It was found in this study that career satisfaction, involvement in challenging case discussions, marital status, gender, perceived advantages of promotion and professional title all significantly influenced the level of work engagement.

It was also found in this study that the higher the career satisfaction, the higher the level of work engagement among specialist nurses, which was consistent with previous research conducted by Wang et al [23]. Career satisfaction was intricately linked to work engagement, indicating the availability of adequate external resources. Work satisfaction serves as a holistic indicator of individual's dedication and rewards for their work. Factors that contribute to the work satisfaction of specialist nurses include social cohesion within the team, professional autonomy, career development, positive social interactions and work environment, as well as effective leadership [28]. Work satisfaction among specialist nurses can be enhanced by professional development, training, accumulated experience, involvement in problem-solving and work autonomy. Nurses who experience high levels of satisfaction tend to feel satisfied and accomplished with their work, fostering a positive attitude and motivation to engage more deeply in their work.

The results of the study showed that specialist nurses who are involved in challenging case discussions exhibited high levels of work engagement. These cases, characterized by complex care issues or multidisciplinary challenges, require collaborative efforts to resolve [29]. Successfully addressing these challenges not only bring a sense of satisfaction and achievement to the specialist nurses but also fuel their motivation to demonstrate increased levels of dedication and commitment to their work. By active involvement in challenging case discussions and fulfillment of their clinical responsibilities, nurses not only accelerate patient recovery but also bolster their overall well-being. Furthermore, these interactions reinforce the specialist nurses' sense of purpose and dedication to their work. Moving forward, it is imperative to actively facilitate challenging case discussions to encourage the involvement of nurses from various specialties, as this approach will not only provide ways for personal growth and professional development but also enable nurses to leverage their expertise effectively and collaborate on finding solutions collectively, thereby ultimately promoting the professional advancement of individual nurses within their specialties and facilitating healthcare teamwork and patient outcomes [30].

It was found in this study that married specialist nurses exhibited higher levels of work engagement, which was consistent with the findings of Arlene et al [31], probably because the multiple roles and responsibilities that married specialist nurses juggled, such as being a spouse and a parent, which could result in increased dedication to their work [32]. Furthermore, with the backing of their families, as well as accumulated age and professional experience, married specialist nurses could adapt positively to their work environment. As the understanding of specialist nurses grows, their work engagement is likely to be strengthened [33]. It is imperative for nursing managers to take the family situation of this group into account and offer appropriate support and resources to enhance the commitment levels of specialist nurses to their work.

The results of this study showed that female specialist nurses exhibited higher levels of work engagement compared to male specialist nurses, which contradicted the results of a previous study [20], with the possible reason that Qin Hui's research was conducted in Jiangsu Province while the current study was conducted in Xiamen City, where a majority of specialist nurses were from southern Fujian Province. Women in southern Fujian are often influenced by traditional gender roles that emphasize care giving, leading to a greater investment of emotion and energy in their work. Furthermore, the nursing profession has historically been dominated by women, and male nurses may face challenges in establishing a strong sense of professional identity and commitment due to societal expectations and personal characteristics [34].

Moving forward, it is imperative to enhance the training and support systems for male nurses and nursing professionals, and efforts should be made to increase positive visibility and recognition of male nurses, educate the general public for positive publicity for male nurses, change traditional concepts and establish a more inclusive and diverse perspective on the nursing profession, thereby ultimately enhancing the contribution of male nurses and advance the overall development of the nursing profession.

Specialist nurses who have completed a specialist nurse course experience increased work engagement and career advancement. Promotion serves as recognition of one's abilities and work performance, providing access to more resources and a higher social status [35]. Positive feedback and expectations within a profession can enhance an individual's ability to value available resources and work harder. Praskova and Johnston emphasized [36], based on the future orientation framework that the potential feedback of a career significantly impacted one's sense of meaning and level of work engagement. Therefore, it is suggested that nursing managers should pay attention to perfect the promotion mechanism for specialist nurses in order to motivate and inspire them to actively engage in their work, thereby ultimately enhancing the quality and efficiency of overall nursing services.

One work-related predictor belonging to a specific profile is the professional title held, which is consistent with Yin et al [37]. Charge nurses and associate chief nurses or above have a higher level of work commitment compared to nurse practitioners. Nurses at the associate nurse practitioner level and above often take on leadership roles, such as nurse team leaders and nurse managers, requiring solid professional knowledge, skills and analytical and problem-solving abilities. Consequently, they may exhibit more entrepreneur spirit, responsibility and proficiency than ordinary nurses and require a higher level of commitment to their work. Additionally, advanced practice nurses typically have more resources to work with and greater autonomy in their work, which may contribute to higher levels of work engagement [38, 39]. In contrast, nurses with the title of nurse practitioner are in a stage of professional independence where they are starting to work independently and take on more responsibility and pressure [40]. During this phase, many of them may reassess their position and fit within the nursing profession, potentially leading to a decline in their work commitment.

Hospitals and nursing managers should implement a comprehensive management system for nurse practitioners from a managerial perspective and tailor interventions focusing on enhancing their work engagement. Relevant studies have shown that standardized management of specialist nurses and investment in the development of specialist nurses can greatly enhance their effectiveness, and management support for specialist nurses has a positive impact on their development [41–43]. Therefore, it is recommended that hospital managers pay attention to the working environment and working conditions of specialist nurses and create better working conditions for specialist nurses by reducing the workload and optimizing the work schedule [44]. In addition, it is also recommended that hospital management, nursing departments and department managers work together to elevate the professional recognition of specialist nurses, establish clear career advancement pathways, offer career planning guidance, implement effective performance evaluation and incentive structures, and provide ample hospital support to foster a sense of engagement and commitment to work among nurses, thereby increasing their work commitment, and ultimately achieving high-quality development for both the hospital and the specialist nurses.

### Limitation

There are also several limitations in this study. Firstly, the results should be interpreted with caution due to the limited representativeness of the sample and the inability to establish causality in cross-sectional studies. Secondly, this study focused solely on specialist nurses in Xiamen, China, which may introduce regional bias. Specialist nurses from a more diverse range of regions should be included in future studies to mitigate potential bias generated by regional differences. Furthermore, only fewer factors are statistically significant in the study, which may be attributed to the multifaceted nature of work engagement of nurses. Various factors such as personal characteristics, work environment and organizational culture can influence work engagement of nurses, and the interactions between these factors are complex. As a result, certain factors may appear more prominently in statistical analysis while others are overlooked. Additionally, subjective perceptions and attitudes towards work engagement of individual nurses play a significant role in influencing their levels of engagement. Quantitative research may not fully capture these subjective factors. Therefore, complex mechanisms and individual experiences behind nurses' work engagement may be considered in future studies to complement quantitative studies and enhance the depth and comprehensiveness of the studies.

## Conclusion

Specialist nurses in Xiamen, China exhibit a high level of work engagement, with notable differences in career satisfaction, involvement in challenging case discussions, marital status, gender, promotion opportunities, and professional title. As such, it is imperative for the government to formulate relevant policies and provide scientific and reasonable support such that specialist nurses can have greater autonomy and room for growth in their profession. Medical institutions should improve the management system for specialist nurses, create dedicated full-time positions, define clear job responsibilities, establish effective performance evaluation and incentive mechanisms, foster nursing research, offer diverse promotion pathways, and cultivate a strong sense of ownership among specialist nurses. Meanwhile, there is a need to strategically plan the workload of nursing staff, streamline workflows, enhance the working environment, prioritize training for male and unmarried specialist nurses, and focus on the development of nurses with lower professional titles. By taking measures to enhance the work engagement of specialist nurses and supporting their professional development, the quality of care and services throughout the hospital may be improved.

## Relevance to clinical practice

There are several implications for practice in this study. A sound and professional title evaluation system should be established for specialist nurses to allow specialist nurses to obtain titles appropriate to their abilities, obtain relevant benefits and take on corresponding responsibilities at an earlier stage in their careers, which can significantly enhance the work engagement of specialist nurses.

In consideration with the significance of enhancing work engagement among male nurses and unmarried nurses, it is recommended that hospital managers take unique characteristics of these groups into account and tailor specific training programs to meet their needs.

Career satisfaction is essential for specialist nurses to be fully engaged in their work. However, when specialist nurses face high levels of role stress and strain due to the demands of their clinical work, diminished enthusiasm and decreased work engagement are ultimately caused. Therefore, workload distribution and benefits should be considered to achieve effective management.

Hospitals and nursing managers should actively engage in a nursing management program that is supported by legal and institutional frameworks, which will ensure adequate capacity to effectively enhance the involvement of specialist nurses, thereby enabling specialist nurses to better serve their patients and ultimately improve the competitiveness of the hospital.

### Supplementary Information

Supplementary Material 1. 

## Data Availability

The datasets generated and analyzed during the current study are not publicly available due to privacy protection and ethical considerations but are available from the corresponding author upon reasonable request.

## References

[1] Tomietto M, Paro E, Sartori R, Maricchio R, Clarizia L, De Lucia P, Pedrinelli G, Finos R (2019). Work engagement and perceived work ability: an evidence-based model to enhance nurses' well-being. J Adv Nurs.

[2] Wan Q, Zhou W, Li Z, Shang S (2018). Associations of organizational justice and job characteristics with work engagement among nurses in hospitals in china. Res Nurs Health.

[3] Ruichen L, Kejimu S, Liangnan Z, Wei Y, Qianqian L, Ying X, Cheng L, Shenhong Z, Daiying Z (2023). Evaluation of work engagement and its determinants in operating room specialist nurses in sichuan province. J Nurs Sci.

[4] Zhang X, Jiang C, Chen F, Wu H, Yang L, Jiang Z, Zhou J (2023). Icu quasi-speciality nurses' knowledge, attitudes and practices regarding early mobilization: a cross-sectional survey. Nurs Open.

[5] Lukewich J, Asghari S, Marshall EG, Mathews M, Swab M, Tranmer J, Bryant-Lukosius D, Martin-Misener R, Norful AA, Ryan D, Poitras ME (2022). Effectiveness of registered nurses on system outcomes in primary care: a systematic review. BMC Health Serv Res.

[6] Markle-Reid M, Mcainey C, Fisher K, Ganann R, Gauthier AP, Heald-Taylor G, Mcelhaney JE, Mcmillan F, Petrie P, Ploeg J, Urajnik DJ, Whitmore C (2021). Effectiveness of a nurse-led hospital-to-home transitional care intervention for older adults with multimorbidity and depressive symptoms: a pragmatic randomized controlled trial. Plos One.

[7] ICN.Nurse practitioner/advanced practice nursing network:definition and characteristics of role[EB/OL].(2018-12-29)[2022-03-29]. http://icn-apnetwork.org/.

[8] Dong Z, Wei L, Sun X, Xiang J, Hu Y, Lin M, Tan Y (2023). Experiences of nurses working in nurse-led clinics in traditional chinese medicine hospitals: a focused ethnographic study. Nurs Open.

[9] M. Wright, TA. Kvist SM. Imeläinen KS. Jokiniemi. Continuing education for advanced practice nurses: a scoping review. J Adv Nurs. (2023). 10.1111/jan.15911. 10.1111/jan.15911 37902130

[10] Farrell C, Molassiotis A, Beaver K, Heaven C (2011). Exploring the scope of oncology specialist nurses' practice in the uk. Eur J Oncol Nurs.

[11] Kapra O, Asna N, Amoyal M, Bashkin O, Dopelt K (2023). The oncology clinical nurse specialist: a rapid review of implementation models and barriers around the world. Curr Oncol..

[12] Martsolf GR, Gigli KH, Reynolds BR, Mccorkle M (2020). Misalignment of specialty nurse practitioners and the consensus model. Nurs Outlook.

[13] Kunie K, Kawakami N, Shimazu A, Yonekura Y, Miyamoto Y (2017). The relationship between work engagement and psychological distress of hospital nurses and the perceived communication behaviors of their nurse managers: a cross-sectional survey. Int J Nurs Stud.

[14] Van Bogaert P, van Heusden D, Timmermans O, Franck E (2014). Nurse work engagement impacts job outcome and nurse-assessed quality of care: model testing with nurse practice environment and nurse work characteristics as predictors. Front Psychol.

[15] García-Iglesias JJ, Gómez-Salgado J, Ortega-Moreno M, Navarro-Abal Y (2020). Relationship between work engagement, psychosocial risks, and mental health among spanish nurses: a cross-sectional study. Front Public Health.

[16] Zhang M, Zhang P, Liu Y, Wang H, Hu K (2021). Du M, Influence of perceived stress and workload on work engagement in front-line nurses during covid-19 pandemic. J Clin Nurs.

[17] Wang Y, Gao Y, Xun Y (2021). Work engagement and associated factors among dental nurses in china. BMC Oral Health.

[18] Zhang L, Bu P, Liu H (2023). Work engagement, emotional disorders and conflict management styles in paediatric nurse: a mediating effect model. Nurs Open.

[19] Y. Kato, R. Chiba, A. Shimazu, Y. Hayashi, T. Sakamoto. Antecedents and outcomes of work engagement among psychiatric nurses in japan. Healthcare. 2023; 11 (3). 10.3390/healthcare11030295. 10.3390/healthcare11030295 PMC9914315 36766870

[20] Qin H, Wu J, Wang T, Wu J, Zhou J (2022). The level and determinants of work engagement among nurse specialists in jiangsu province. J Nurs Sci.

[21] von Elm E, Altman DG, Egger M, Pocock SJ, Gøtzsche PC, Vandenbroucke JP (2007). The strengthening the reporting of observational studies in epidemiology (strobe) statement: guidelines for reporting observational studies. Ann Intern Med.

[22] Qin H, Wu J, Wang T, Wu J, Zhou J (2022). Development and psychometric testing of the specialty nurse work engagement scale. J Nurs Sci.

[23] Wang Y, Tang L, Li L (2023). Work engagement and associated factors among healthcare professionals in the post-pandemic era: a cross-sectional study. Front Public Health.

[24] Schaufeli W, Salanova M (2006). The measurement of work engagement with a short questionnairea cross-national study. Educ Psychol Meas.

[25] Yanming D, Xinjuan W, Junye T, Peiying Z, Yanyan X, Xiuying W (2021). The current situation of the cultivation and development of specialty nurses in tertiary hospitals in china. Chin J Nurs.

[26] Hofmeyer A, Taylor R (2021). Strategies and resources for nurse leaders to use to lead with empathy and prudence so they understand and address sources of anxiety among nurses practising in the era of covid-19. J Clin Nurs.

[27] Vargas-Benítez MÁ, Izquierdo-Espín FJ, Castro-Martínez N, Gómez-Urquiza JL, Albendín-García L, Velando-Soriano A, Cañadas-De LFG (2023). Burnout syndrome and work engagement in nursing staff: a systematic review and meta-analysis. Front Med.

[28] C. Foà, M.C. Guarnieri, G. Bastoni, B. Benini, O.M. Giunti, M. Mazzotti, C. Rossi, A. Savoia, L. Sarli, G. Artioli, Job satisfaction, work engagement and stress/burnout of elderly care staff: a qualitative research, Acta Biomed. 2020; 91(12-S); e2020014. 10.23750/abm.v91i12-S.10918. 10.23750/abm.v91i12-S.10918 PMC8023104 33263342

[29] Yuan Y, He S, Huang J, Li D, Zhang H (2015). Multidisciplinary discussion of difficult and complicated nursing cases through workshop activity. J Nurs Sci.

[30] Slatyer S, Coventry LL, Twigg D, Davis S (2016). Professional practice models for nursing: a review of the literature and synthesis of key components. J Nurs Manag.

[31] A. Pericak, C.W. Hogg, K. Skalsky, L. Bourdeanu, What influences work engagement among registered nurses: implications for evidence-based action, Worldviews Evid.-Based Nurs. 2020. 10.1111/wvn.12469. 10.1111/wvn.12469 33090622

[32] Okada N, Yabase K, Kobayashi T, Okamura H (2019). Do multiple personal roles promote working energetically in female nurses? A cross-sectional study of relevant factors promoting work engagement in female nurses. Environ Health Prev.

[33] Balay-Odao EM, Cruz JP, Alquwez N, Al OK, Al TA, Alotaibi RS, Valencia JA, Danglipen CC (2022). Structural empowerment and work ethics influence on the work engagement of millennial nurses. J Nurs Manag.

[34] Bumbach MD, Harman JS, Lucero R, Cimiotti JP, Felber ND (2020). Gender differences in nurse practitioners: job satisfaction and patterns of care. J. Am. Assoc. Nurs. Pract..

[35] Song M, Zheng Q, Jiang A, Cai M, Gao Y. Leadership coaching and subordinate engagement: a self-determined perspective. Manag Rev. 2023; 35(5):163-172. 10.14120/j.cnki.cn11-5057/f.2023.05.017.

[36] Praskova A, Johnston L (2020). The role of future orientation and negative career feedback in career agency and career success in australian adults. J Career Assess.

[37] Yin Y, Lyu M, Zuo M, Yao S, Li H, Li J, Zhang J, Zhang J (2022). Subtypes of work engagement in frontline supporting nurses during covid-19 pandemic: a latent profile analysis. J Adv Nurs.

[38] Bamford M, Wong CA, Laschinger H (2013). The influence of authentic leadership and areas of worklife on work engagement of registered nurses. J Nurs Manag.

[39] Wan Q, Zhou W, Li Z, Shang S, Yu F (2018). Work engagement and its predictors in registered nurses: a cross-sectional design. Nurs. Health Sci.

[40] Keyko K, Cummings GG, Yonge O, Wong CA (2016). Work engagement in professional nursing practice: a systematic review. Int J Nurs Stud.

[41] Dempsey C, Assi MJ (2018). The impact of nurse engagement on quality, safety, and the experience of care: what nurse leaders should know. Nurs Adm Q.

[42] Kilpatrick K, Tchouaket E, Carter N, Bryant-Lukosius D, Dicenso A (2016). Structural and process factors that influence clinical nurse specialist role implementation. Clin Nurse Spec.

[43] Cai Y, Li Q, Cao T, Wan Q (2023). Nurses' work engagement: the influences of ambidextrous leadership, clinical nurse leadership and workload. J Adv Nurs.

[44] Luo Y, Feng X, Wang D, Qiao X, Xiao X, Jia S, Zheng M, Reinhardt JD (2023). Experience of clinical nurses engaged in caring for patients with covid-19: a qualitative systematic review and meta-synthesis. J Clin Nurs.

